# Cardiovascular disease in children in Djibouti: a single-centre study

**DOI:** 10.11604/pamj.2013.14.141.2016

**Published:** 2013-04-09

**Authors:** Pierre-Laurent Massoure, Nicolas-Charles Roche, Gatien Lamblin, Celine Dehan, Eric Kaiser, Laurent Fourcade

**Affiliations:** 1 Hôpital Laveran, 13 384 Marseille cedex 13, France; 2 Hôpital Bouffard, Djibouti, SP 85024, 00812 Armées

**Keywords:** Congenital heart disease, cardiac surgery, dilated cardiomyopathy, Horn of Africa

## Abstract

**Introduction:**

Few data are available about pediatric cardiovascular disease (CVD) in the Horn of Africa. The objective of this study was to describe the spectrum of CVD in children in Djibouti.

**Methods:**

Clinical features and management of Djiboutian children between 1 month-old and 15 year-old with CVD were prospectively recorded over a two-year period in Bouffard Military Hospital in Djibouti (January 2009- December 2010).

**Results:**

Clinical examination and echocardiography were performed on 156 patients: 32 of them (20%) had CVD. Three (10%) of them had Down's syndrome. The median age was 5 years (male 53%). Congenital heart disease was observed in 27 (84%) patients and dilated cardiomyopathy (DCM) in 5 (16%) patients including 2 patients with rheumatic valvular disease. Ventricular septal defect was frequent (28%). Other abnormalities were atrial septal defect (13%), Tetralogy of Fallot (9%), pulmonary stenosis (6%) and 3 other patients had multiple congenital anomalies condition. Surgical management was required in 22 (69%) patients and was performed on 15 (47%) cases. During follow up (mean 11.3 ± 6.8 months), 5 (16%) patients died. Absence of surgery was associated with significant mortality (p > 0.05) but age, sex and mean follow up were not.

**Conclusion:**

Pediatric CVD is at least as common in this Djiboutian community as in other African cohorts. The absence of surgery was a major mortality risk factor. DCM was frequent in this study. Much work remains to be done to discover the size and nature of genetic and environmental contributions to these various forms of heart diseases in the Horn of Africa.

## Introduction

Cardiovascular disease (CVD) constitutes a major burden in developed and developing countries. Hundreds of thousands of children die each year from congenital heart diseases (CHD), while millions more remain in desperate need of treatment in the developing world. CHD represent a large part of CVD during the first years of life but little is known about the incidence and patterns of CVD and CHD in the Horn of Africa [1–3]. CHD is one of the most frequent birth defect with a live-born prevalence of about 0.5 to 0.8% up to 2% in African systematic echocardiography series [4]. In Djibouti, the spectrum of CHD and acquired cardiomyopathy in children is not known. The objective of this study was to describe the spectrum of CVD and to determine the risk factors of mortality in children in a Djiboutian single-centre study.

## Methods

Consecutive children were addressed to the cardiology unit of Military Hospital Bouffard (Djibouti) for cardiac symptoms or cardiac murmur, and were prospectively studied over a 2 two-year period (January 2009 - December 2010). Their age ranged between 1 month and 15year-old. Patients were addressed from local medical centres, other hospitals in Djibouti or were directly hospitalized in our centre. Clinical data included age, gender, symptoms (such as breathlessness, repeated chest infections) and clinical findings (cardiac murmur, cyanosis). Human Immunodeficiency Virus (HIV) status was noted. Children with CHD and children with other inherited or acquired cardiomyopathy were included. Patent ductus arteriosus (PDA) and patent foramen ovale in children under three month-old were not considered as CHD except in case of a large PDA.

Echocardiographies of all patients were performed according to the recommendations of current guidelines with a Siemens-Acusson CV 70 cardiovascular system (P 4-2 MHz adult transducer and P 9-4 MHz pediatric transducer) [5]. Results from other imaging examinations, including Computed Tomography Scanner were also obtained.

Outcome and management pathway were noted. There was no possibility for cardiac surgery in Djibouti. When it was required and possible, CHD were sent abroad for cardiac surgery. Type of surgery and outcome after surgery were reported.

The group of survivors at the end of follow up was compared to the group of non-survivors at the end of follow up. Mortality risk factors were analyzed. For statistical analysis, Microsoft Excel^®^ software was used. Data were expressed as mean ± standard deviation (range). Discrete variables were presented as percentages. Data were analysed by Student's t test for continuous variables or in two-by-two tables using the Chi-square'test for categorical variables. A p value < 0.05 was accepted as significant. A p value ≥ 0.05 was not significant (NS).

## Results

Over a 20 months period, 156 children were addressed for presumed CVD and had a complete examination and echocardiography. Among them, 32 (20%) had CVD. The mean age at referral was 5.8 ± 4.9 year-old with a median of 5 years (0.1 to 15 years) in patients with CVD. Seven (22%) children were less than 1 year-old. Seventeen (53%) were male. Symptoms and clinical signs are summarized in Table 1. Distribution of CVD is summarized in Table 2. Twelve (37%) patients had ventricular septal defect (VSD). Six (19%) patients had isolated ventricular septal defect (VSD), membranous in 4 cases, muscular in 2 cases. The size of VSD was small in all these 6 cases (3 mm or less). Six patients had large VSD associated with other abnormalities: 3 (9%) cases of complex CHD (surgical repair was performed on 2 cases - see Table 2 for details), and in 3 (9%) cases of tetralogy of Fallot (TOF) with a favourable outcome after successful surgery.

**Table 1:** Clinical signs and symptoms in 32 Djiboutian children with cardiovascular disease

Clinical signs and symptoms	n (%)
Cardiac murmur	26 (81%)
Dyspnea	25 (78%)
Retardation of growth	14 (43%)
Repeated pulmonary infections	9 (28%)
Cyanosis	7 (22%)
Syncope	3 (10%)

**Table 2:** Distribution, management and outcome of cardiovascular disease in 32 Djiboutian children

Diseases	n	Male	Surgery required	Surgery performed	Non survivors
**Isolated membranous VSD**	**6**	**5**	**0**	**0**	**0**
VSD + ASD (OS) + PDA {DS}	1	1	1	1	0
VSD + ASD (OS) {DS}	1	0	1	1	0
VSD + double outlet right ventricle	1	1	1	To be offered	0
Tetralogy of Fallot	3	2	3	3	0
PDA {1 DS}	2	0	2	1	1*
ASD (OS) + PDA	2	0	2	1	1*
Isolated ASD (OS)	2	1	2	1	0
Pulmonary stenosis	2	2	2	2	0
Congenitally corrected TGV	2	1	0	0	0
**Others**					
Cor triatriatum	1	0	1	0	0
Complete Atrioventricular canal defect	1	0	1	1	0
Single ventricle + hypoplasic pulmonary	1	0	1	1	0
Trunk + anomalous venous (azygos) return	1	1	1	1	0
Subaortic stenosis Situs inversus + Pulmonary Hypertension	1	0	0	0	0
DCM with severe MR (2 with RVD)	5	3	5	2	3*
**Total**	**32**	**17**	**22**	**15**	**5**

VSD: ventricular septal defect, ASD: atrial septal defect, OS: ostium secundum, PDA: Patent ductus arteriosus, DS: Down syndrome, TGV: transposition of great vessels, DCM: dilated cardiomyopathy, MR: Mitral regurgitation, RVD: rheumatic valvular disease

* died before required

Three (9%) patients (1 male) had Down syndrome. Three (9%) other patients had multiple congenital anomalies condition. A 6 year-old boy had LEOPARD Syndrome (Lentigines, ECG conduction abnormalities, Ocular hypertelorism, pulmonary stenosis, abnormal genitalia, growth retardation, and sensorineural Deafness). A newborn (1 month-old) was admitted in intensive care for edema and dyspnea and had large patent ductus arteriosus (PDA) and ostium secundum atrial septal defect (ASD) associated with kidneys abnormality and renal failure. A 2 year-old girl had the association of a double inlet single ventricle with a large ASD, pulmonary trunk and pulmonary arteries hypoplasia and anomalous venous return (interruption of the inferior vena cava with azygos continuation forming an arch returning into the right superior vena cava).

Five (16%) patients had dilated cardiomyopathy (DCM) with severe mitral regurgitation. Two of them had rheumatic mitral valvular disease with typical echocardiographic feature. Familial screening was offered to the first degree relatives of children with “idiopathic” DCM and revealed one other case of DCM.

Twenty four patients were negative for HIV. The others were not tested (non hospitalized patients). At the end of follow up (11.3± 6.8 months), 27 (84%) patients were survivors (15 males, mean age 5.7 ± 4.8 years). Five (16%) patients were non-survivors (2 males, mean age 6.3± 5.7 years). Three of them had severe mitral regurgitation (MR) in advanced stage. They were admitted in emergency and the CVD was diagnosed at the end stage of heart failure: 2 died during the first hospitalization for heart failure, and one died 2 months before planned cardiac surgery for severe rheumatic mitral valvulopathy. One child with a large PDA died in her village at the age of 8 months with a severe growth retardation, undernutrition and malnutrition. One child with PDA and large ASD associated with renal failure died after one month of treatment in intensive care unit.

Surgical management was required in 22 (69%) children and was performed on 15 (47%) cases (Table 2). Among the 7 other cases, the family of two patients refused the surgical treatment and 5 patients died before surgery. All surgical cases but one had complete surgical repair. A left systemic to pulmonary shunt (palliative surgery) was performed on the 2-year old girl with single ventricle and pulmonary arteries hypoplasia. Surgery was performed on 12 children sent abroad with humanitarian programs (10 in France, 1 in Sudan and 1 in Italy) with a favourable outcome during follow up. Three patients had surgery in India using personal means. All the patients in this cohort are still currently undergoing surveillance except the 2 patients who refused surgical treatment and who were lost of follow up. At the end of follow up, sex, age and mean follow up were not associated with mortality (p = NS). Absence of surgery was associated with mortality (p < 0.05).

## Discussion

This cohort study is the first report about CVD in a Djiboutian pediatric population. It is estimated that about 35% of the 746 000 people living in Djibouti is less than 15 years of age. There are no cardiac catheterization facilities in Djibouti and only 2 centres, including our center, are performing pediatric echocardiography. This small single-centre study was not designed to lead to important epidemiological results.

In patients with CVD, the sex ratio is similar to the results reported in other studies [1, 6–9]. The median age (5 years) at referral was higher in our study than in other series. For example, mean age at diagnosis was 4 years in a Sudanese population with CHD, 2 years in Zimbabwe [1, 7]. This is probably due to the lack of medical facilities in extremely poor population which represents the majority of the included patients. Symptoms and clinical signs were similar to the results observed in other studies in Africa and in other parts of the world [7, 8].

The most common heart disease was VSD, associated or non-associated with other cardiac malformations. VSD accounted for 37% of all the cases in this study. Small VSD is usually benign, frequently decrease in size and 30-50% undergo spontaneous closure. Other reports from Africa had similar findings [1, 7, 9]. The other predominant congenital abnormalities were TOF, ASD and PDA. Some rare findings were reported in this small cohort (Table 2). As shown recently, CHD rather than acquired heart diseases are dominant in many African settings [10, 11]. In a recent study of 507 recruited patients with heart disease in Cameroon, 67% had CHD. Regional differences were noted but isolated ventricular septal defect remained the main type of CHD. Various types of CHD were described such as common arterial trunk, transposition of great arteries with ventricular septal defect, Ebstein disease, coarctation of the aorta, anomalous pulmonary venous return and left isomerism [11]. These particular findings were not reported in this 2 years single-centre study in Djibouti.

Dilated cardiomyopathy was frequent in this study. Two cases of severe mitral regurgitation in advanced stage were a consequence of rheumatic heart disease. MR is the most common finding in children with rheumatic heart disease in the Horn of Africa. MR was found in 84% of children with rheumatic valvular disease in Sudan [12]. Cases of dilated cardiomyopathy were not attributed to HIV related infection. The absence of echocardiographic pattern of endomyocardial fibrosis was not unexpected. This disease has never been described in Djibouti. Heredity and genetic factors, nutritional status and vitamin deficiency, infectious diseases and presumed myocarditis are possible underlying condition in patients with dilated cardiomyopathy [13].

This study did not set out to establish the etiological factors in patients with CHD. However, 3 (9%) patients had Down syndrome associated with various types of CHD (Table 2). Down syndrome is usually found in about 5% of CHD in large African studies [3, 14].

The absence of surgery was a significant mortality factor whereas age and sex were not. This suggests that a significant number of patients may miss the opportunity to have optimal surgical intervention. In other African studies, parental income appears to influence the management pathway, however, the level of parental education and patient sex did not [14]. This study had limitations. This study was a description of Djiboutian patients who were managed in a French military hospital and was necessarily influenced by referral and anti-referral patterns. Genetic analysis and vitamin level analysis were not feasible in patients with DCM. The rate of CVD (N = 32/156 children - 21% of addressed patients) had no epidemiological value but the wide range of children with diverse CVD is a reflection of developing pediatric cardiology facilities locally, with regional and international collaboration.

## Conclusion

This study confirmed that congenital heart pattern is at least as common in this Djiboutian community as in other African cohorts. Ventricular septal defect was the predominant pathology. Age at referral was higher than previously described in other African cohorts. The absence of surgery remained the major mortality risk factor. DCM was frequent in this study. Much work remains to be done to improve the newborn and children medical screening in Djibouti and to discover the size and nature of genetic and environmental contributions to these various forms of heart diseases in the Horn of Africa.

## References

[1] Sulafa KM, Karani Z (2007). Diagnosis, management and outcome of heart disease in Sudanese patients. East Afr Med J..

[2] Marijon E, Tivane A, Voicu S, Vilanculos A, Jani D, Ferreira B, Ou P (2006). Prevalence of congenital heart disease in schoolchildren of sub-Saharan Africa, Mozambique. Int J Cardiol..

[3] Sulafa KM (2009). Cardiac abnormalities of Sudanese patients with Down's syndrome and their short-term outcome. Cardiovasc J Afr..

[4] Sani MU, Mukhtar-Yola M, Karaye KM (2007). Spectrum of congenital heart disease in a tropical environment: an echocardiography study. J Natl Med Assoc..

[5] Quinones MA, Otto CM, Stoddard M (2002). Recommendations for Quantifications of Doppler Echocardiography. A report from the Doppler Quantification Task Force of the Nomenclature and Standards Committee of the American Society of Echocardiography. J Am Soc Echocardiogr..

[6] Bassili A, Mokhtar SA, Dabous NI, Zaher SR, Mokhtar MM, Zaki A (2000). Congenital heart disease among school children in Alexandria, Egypt: an overview on prevalence and relative frequencies. J Trop Pediatr..

[7] Bannerman CH, Mahalu W (1998). Congenital heart disease in Zimbabwean children. Ann Trop Paediatr..

[8] Huber J, Peres VC, dos Santos TJ, Beltrão L, de Baumont AC, Cañedo AD (2010). Congenital heart diseases in a reference service: clinical evolution and associated illnesses. Arq Bras Cardiol..

[9] Diop IB, Ndiaye M, Ba SA, Sarr M, Kane A, Hane L (1996). Congenital heart disease surgery in Senegal. Indications, evaluation and perspectives. Dakar Med..

[10] Okoromah CA, Ekure EN, Ojo OO, Animasahun BA, Bastos MI (2008). Structural heart disease in children in Lagos: profile, problems and prospects. Niger Postgrad Med J..

[11] Tantchou Tchoumi JC, Ambassa JC, Chelo D, Djimegne FK, Giamberti A, Cirri S (2011). Pattern and clinical aspects of congenital heart diseases and their management in Cameroon. Bull Soc Pathol Exot..

[12] Alkhalifa MS, Ibrahim SA, Osman SH (2008). Pattern and severity of rheumatic valvular lesions in children in Khartoum, Sudan. East Mediterr Health J..

[13] Mayosi BM, Somers K (2007). Cardiomyopathy in Africa: heredity versus environment. Cardiovasc J Afr..

[14] Awori MN, Ogendo SW, Gitome SW, Ong'uti SK, Obonyo NG (2007). Management pathway for congenital heart disease at Kenyatta National Hospital, Nairobi. East Afr Med J..

